# Short-term effectiveness of gambling treatment in the Daily Clinic for Gambling Addiction

**DOI:** 10.3389/fpsyg.2025.1536082

**Published:** 2025-03-26

**Authors:** Dora Dodig Hundric, Neven Ricijas, Sabina Mandic, Sanja Radic Bursac, Davor Bodor

**Affiliations:** ^1^Department of Behavioural Disorders, University of Zagreb Faculty of Education and Rehabilitation Sciences, Zagreb, Croatia; ^2^Teaching and Clinical Centre, University of Zagreb Faculty of Education and Rehabilitation Sciences, Zagreb, Croatia; ^3^Daily Clinic for Gambling Addiction, University Psychiatric Hospital “St. John”, Zagreb, Croatia

**Keywords:** gambling, gambling disorder, gambling addiction, treatment, evaluation, effectiveness

## Abstract

In response to the high prevalence of gambling addiction within the population, a specialised Daily Clinic for Gambling Addiction was established at the University Psychiatric Hospital “St. John” in Zagreb (Croatia). This clinic offers a unique three-month, semi-structured, intensive multidimensional and multidisciplinary treatment approach delivered by a team of specialised mental health professionals. Treatment interventions include individual and group psychotherapy, socioemotional skills training, family therapy, support groups and other modalities. In collaboration with researchers from the University of Zagreb Faculty of Education and Rehabilitation Sciences, the study team is conducting a scientific evaluation to determine the outcomes of the treatment. This study presents results on its’ short-term effectiveness, based on a sample of *N* = 209 patients (Mean Age = 33.54; Males = 92.8%; Females = 7.2%) who underwent treatment between 2017 and 2021. To assess the effectiveness of the treatment, a research design incorporating two measurement sessions (pre-test and post-test) was employed. This design utilised a comprehensive battery of validated instruments, each targeting specific constructs or domains of psychosocial functioning that the intervention aims to address. The assessment tools included: (1) Problem Gambling Severity Index-PGSI, (2) Gambling Attitudes Scale—GAS, (3) Coping Inventory for Stressful Situations—CISS, (4) The Gambling Beliefs Scale—short version, (5) Problem Solving and Refusal Skills Scale, (6) Depression, Anxiety, Stress Scale—DASS-21, (7) Generalised Self-Efficacy Scale—GSE, and (8) Multidimensional Scale of Perceived Social Support. The results indicate significant positive improvements in gamblers’ psychosocial functioning with the following large effect sizes: gambling-related consequences (*r* = 0.84), task oriented coping (Cohen’s *d* = 0.79), emotion oriented coping (Cohen’s *d* = 1.06), attitudes (*r* = 0.67), superstition (*r* = 0.61), illusion of control (*r* = 0.62), depression (*r* = 0.78), anxiety (*r* = 0.71), stress (*r* = 0.73), problem-solving skills (*r* = 0.73) and general self-efficacy (*r* = 0.61). The effects on refusal skills (Cohen’s *d* = 0.48) as well as on alcohol (Cohen’s *d* = 0.24) and marihuana (*r* = 0.26) were small to medium. Findings are discussed in terms of appropriate treatment approaches for gambling addiction, methodological challenges in measuring effects and implications for future evaluation research. In general, this treatment protocol provides promising effects for gambling addiction.

## Introduction

Gambling is a phenomenon that is prevalent across all age groups, and the gambling industry has been one of the fastest-growing industries worldwide in recent decades (Raspor et al., 2019; Grande-Gosende et al., 2019; Ricijaš, 2020; Sulkunen et al., 2020). The development of digital technology and the liberalisation of the gambling market in some countries have certainly contributed to the increasing availability and accessibility of gambling (Newall et al., 2019; Dodig Hundric et al., 2021; Sulkunen et al., 2020). It is therefore not surprising that the prevalence of gambling (especially online modalities in recent years) and gambling-related problems are increasing (Bruneau et al., 2016; Bodor et al., 2018; Meyer et al., 2018; Winters and Derevensky, 2019; Skelin and Puljić, 2022). Results of international studies show that around 70% of the adult population gambles at least once a year, while rates of problem gambling range from 0.12 and 5.8% worldwide and between 0.12 and 3.4% in Europe (Calado and Griffiths, 2016; WHO, 2020; Gavriel-Fried et al., 2021). In Croatia, characterized by a highly liberal gambling market (Ricijaš et al., 2020; Šimović et al., 2019), a study conducted on a representative sample of its citizens (*N* = 4,992) aged 15–64 years, indicated that approximately 60% of respondents reported having gambled at least once in their lifetime, with 33.4% having gambled within the past year, and 20.5% within the past month (Glavak Tkalic et al., 2017). In terms of gambling-related problems, it was found that about 9% of the Croatian population met the criteria for gambling-related psychosocial problems (4.3% low, 2.9% moderate and 2.3% severe problems) (Glavak Tkalic et al., 2017). Hence, it is not surprising that meta-analyses identify Croatia as one of the leading Western countries in terms of gambling prevalence and the occurrence of gambling addiction symptoms (Sussman et al., 2010; Lorains et al., 2011; Calado et al., 2017).

Gambling addiction causes, or is correlated with, a whole range of adverse psychosocial consequences such as mental health problems (depression, anxiety, insomnia, irritability), loss of (self-)control and chasing losses, subjective feelings of guilt, hiding gambling from others, development of tolerance and excessive behaviour, conflicts and financial problems caused by gambling. Furthermore, it affects not only the individual but also other people in the addict’s immediate and wider social environment such as partners, children, other family members, friends, business colleagues, acquaintances, etc. (Griffiths, 2003, 2009; Kalischuk et al., 2006; Downs and Woolrych, 2010; Hing et al., 2013; Goodwin et al., 2017; Bodor, 2018; Landon et al., 2018; Latvala et al., 2019; Booth et al., 2021; Globan et al., 2021; Mide et al., 2023). Moreover, macroeconomic research highlights substantial social expenses attributable to gambling addiction, including costs associated with gambling-related crime, labour and employment, bankruptcies, healthcare expenditures, impacts on the welfare system, familial costs, and misallocation of funds (Grinols, 2011; Makarovič et al., 2011; Latvala et al., 2019; Globan et al., 2021). Given the above, gambling addiction has become a widespread and comprehensive public health problem worldwide (Derevensky et al., 2003; Griffiths, 2003; Messerlian et al., 2005), which accordingly requires comprehensive actions within various systems and at all levels of intervention, especially when it comes to the treatment of adverse gambling-related consequences.

In addition to pharmacological therapy, the treatment of gambling addiction is mainly concerned with psychological treatment models, which include various psychotherapeutic, psychosocial and psychoeducational approaches that can be individual, group or combined, as well as structured and non-structured (Jiménez-Murcia et al., 2007; Carlbring et al., 2010; Bodor et al., 2021). Psychotherapeutic methods often employ techniques proven effective in treating substance use disorders, including behavioural and cognitive therapies, motivational interviewing, various self-help strategies, and psychodynamic, psychoanalytic, and multimodal interventions (Blank et al., 2021). Regardless of the therapeutic approach, achieving abstinence from gambling is universally recognised as a critical objective essential for the psychosocial recovery of individuals with gambling addiction (Echeburúa et al., 2000).

In terms of the preferred gambling treatment, cognitive behavioural therapy/treatment (CBT) often stands out due to its robust evidence-based effectiveness compared to other modalities (Fong, 2005; Gooding and Tarrier, 2009; Carlbring et al., 2010; Cowlishaw et al., 2012; Rizeanu, 2015; Abbott et al., 2017; Petry et al., 2017; Garcia-Caballero et al., 2018; Bodor et al., 2021; Diaz-Sanahuja et al., 2021). Furthermore, meta-analytical findings from 25 studies emphasise that the group-based approach demonstrates the most significant long-term benefits (Gooding and Tarrier, 2009). It offers unique therapeutic advantages not found in individual approaches, leveraging social dynamics such as peer pressure, mutual support, and shared learning experiences. These group processes, including mirroring, resonance, and translation of experiences, foster corrective behaviours and enhance self-esteem and self-efficacy among participants (Stojnić, 2018).

Furthermore, treatment strategies for substance use disorders have increasingly incorporated principles of Motivational Interviewing (MI). Empirical evidence suggests that MI is effective in reducing addictive behaviours, enhancing treatment retention, and extending periods of abstinence, particularly when used in combination with other therapeutic modalities such as Cognitive Behavioural Therapy (CBT) (Carroll et al., 2006; Smedslund et al., 2011; Sayegh et al., 2017). Consequently, MI is being increasingly applied in the treatment of gambling disorders. Research has corroborated its efficacy, indicating that MI is a promising intervention (Forsberg et al., 2008, as cited in Carlbring et al., 2010), especially in achieving short-term outcomes (Carlbring et al., 2010; Yakovenko et al., 2015).

Regardless of the implementation approach, almost all psychological treatment models focus on similar factors relevant for positive treatment outcomes. These include cognitive restructuring (addressing cognitive distortions and misconceptions about gambling), identifying alternative behaviours to replace gambling, strengthening communication, social–emotional and problem-solving skills, promoting financial literacy, implementing relapse prevention strategies (such as recognising triggers and coping with stressors) (Sylvain et al., 1997; Ladouceur et al., 2003; Petry, 2005; Dowling et al., 2006; Jiménez-Murcia et al., 2007; Rizeanu, 2015; Garcia-Caballero et al., 2018), as well as involving significant others in the treatment process (Fong, 2005; Dowling et al., 2006; Jiménez-Murcia et al., 2007).

Although there are dozens of developed gambling treatments, there is still a lack of high-quality research that would provide answers about their short-and long-term effects and insights into specific treatment elements that influence their effectiveness (Ladouceur et al., 2003; Toneatto and Ladoceur, 2003; Petry, 2005; Petry et al., 2006; Jiménez-Murcia et al., 2007; Carlbring et al., 2010).

One of the first (semi-)structured treatments based on a cognitive-behavioural approach was developed in 1997 (Sylvain et al., 1997) and its central element is addressing misconceptions about gambling. The authors conducted an outcome evaluation on a sample of *N* = 29 participants and the results demonstrated effectiveness (86% of participants no longer met DSM-III-R criteria, there was an improved perception of their own gambling-related problems, etc.), even after a 12-month follow-up (Sylvain et al., 1997). Several years later, Ladouceur et al. (2001) adapted the programme and conducted a subsequent evaluation involving a sample of *N* = 64 participants. They observed positive effects of individual interventions. Subsequently, in 2003, they implemented a 10-session group programme (120 min each session) which also yielded significant positive changes (Ladouceur et al., 2003).

Dowling et al. (2006) developed a CBT treatment consisting of 12 weekly sessions lasting 90 min, adaptable for both individual and group settings. The authors conducted an evaluation involving a total of *N* = 56 female participants, divided into three groups: (1) *n* = 25 in the control group, (2) *n* = 14 receiving individual treatment, and (3) *n* = 17 participating in group treatment (Dowling et al., 2007). Baseline and six-month follow-up assessments were conducted for all participants. Due to the treatment’s diverse modalities and complexities, the duration varied; group treatment was designed for completion within 12 weeks, while individual treatment durations ranged from 12 to 56 weeks. The findings indicated that the most significant positive changes occurred with the longest durations of individual treatment (Dowling et al., 2007).

Furthermore, Jiménez-Murcia et al. (2007) developed a CBT treatment based on an integrative model (Sharpe and Tarrier, 1993; according to Jiménez-Murcia et al., 2007). Their evaluation conducted with a sample of *N* = 290 gambling addicts demonstrated the programme’s effectiveness in achieving gambling abstinence among 76.1% of patients by the end of the treatment and 81.5% at the six-month follow-up. However, a methodological limitation noted was that up to 50% of patients from the initial sample did not participate in the final follow-up assessment (Jiménez-Murcia et al., 2007).

It is undeniable that the presented interventions achieve certain positive outcomes in terms of reducing gambling activity and improving the individual’s general psychosocial functioning. However, they are not comprehensive, i.e., they do not address the problem from multiple perspectives and therefore, as confirmed by meta-analyses (Rash and Petry, 2014; Petry et al., 2017), do not have sufficient impact on consistent or persistent/long-term changes in the addict’s gambling behaviour and psychosocial functioning. Considering that gambling is a multidimensional problem affecting both the individual and their significant others, it is necessary to address it comprehensively and from multiple perspectives, but also in a way that includes more intensive individual and group treatment, involvement of significant others, etc. Based on the presented evidence, it can be concluded that, despite individual variations among those with gambling addiction, psychosocial interventions should prioritise the following areas: (1) specific personality traits such as impulsivity, excitability, and low frustration tolerance; (2) gambling-related cognitive distortions; (3) socio-emotional skills, including decision-making, problem-solving, critical thinking, and coping mechanisms; (4) concurrent mental health and behavioural problems; and (5) improving social functioning and relationships with significant others, which are often substantially impaired throughout the progression of addiction.

To address the high prevalence of gambling problems in Croatia, a comprehensive, evidence-based outpatient treatment programme for gambling addiction was developed and implemented at the Daily Clinic for Gambling Addiction at the University Psychiatric Hospital “Sveti Ivan [St. John]” in Zagreb. The clinic’s treatment approach is based on the positive effects of group psychotherapy in the treatment of various types of addiction as well as on the principles of therapeutic community, with an emphasis on group work. The programme’s development was guided by established best practice examples and the standards of effective psychosocial interventions (Nation et al., 2003; Starcevic Kaic et al., 2020).

It aims to facilitate stable, long-term abstinence through lifestyle modifications. Regarding working modalities, a multifaceted approach is employed, emphasising psychoeducation, counselling, and both individual and group psychotherapy. This strategy recognises that integrating various therapeutic methods is essential to achieve treatment objectives and facilitate behavioural change. By educating patients about gambling behaviour and cognitive distortions, explaining the development of gambling addiction, encouraging critical thinking and improving skills crucial to maintaining abstinence, patients are empowered to take responsibility for their actions and actively participate in changing the behavioural patterns that contributed to their addiction. By recognising that achieving abstinence is only the first phase of their treatment, which is insufficient itself, the programme focuses on maintaining that abstinence. This is practised through specific short-term goals, such as strengthening intrinsic motivation for active engagement in treatment, promoting self-awareness and a critical attitude towards addiction, improving interpersonal relationships, expanding support networks and improving functioning in the family, workplace/school, and social environment.

Therefore, the aim of this study is to explore the short-term effectiveness of treatment in the Daily Clinic for Gambling Addiction, which represents the first scientific evaluation of this intervention. It also aims to contribute to the scientific knowledge on the effectiveness of different treatment interventions in clinical settings and to, potentially, overcome previous shortcomings.

## Methods

### Intervention

This study examines the short-term effectiveness of the outpatient gambling treatment programme in the Daily Clinic for Gambling Addiction in University Psychiatric Hospital “St. John” (Zagreb, Croatia).

As previously mentioned, this treatment is based on empirical evidence on predictors as well as symptoms of gambling involvement and gambling addiction, already established effective gambling treatment efforts and is consistent with the evidence-based principles of semi-structured psychosocial interventions (Sylvain et al., 1997; Ladouceur et al., 2003; Nation et al., 2003; Petry, 2005; Dowling et al., 2006; Jiménez-Murcia et al., 2007; Rizeanu, 2015; Garcia-Caballero et al., 2018; Starcevic Kaic et al., 2020). In total it lasts 3 months, every working day for 4 h. Given its comprehensiveness, it is carried out by a multidisciplinary team of trained mental health professionals with experience in gambling addiction as well as developing and implementing psychosocial/psychoeducative interventions (medical doctors—psychiatrists, psychologists, social pedagogues, and nurses). The treatment consists of individual and group psychotherapy, interactive psychoeducational workshops, cognitive-behavioural therapy, multifamily therapy, member-led support groups (stable abstainers), and psychopharmacotherapy as needed. A detailed description of the treatment components is provided in Table 1.

**Table 1:** Structure of the treatment in the Daily Clinic for Gambling Addiction (University Psychiatric Hospital “St. John”).

Treatment component	Implementation dynamics	Treatment provider	Treatment component description
Group psychotherapy	Every working day	Psychiatrist, social pedagogue, nurse	By employing diverse evidence-based psychotherapeutic approaches and techniques—including cognitive-behavioural therapy, reality therapy, psychodynamic therapy, systemic family therapy, and motivational interviewing, patients gain knowledge and cultivate interpersonal skills crucial for fostering lasting change beyond the structured clinic environment. Conducted in a group setting designed to provide safety and stability, the treatment utilises a variety of well-established group interactions and experiences.
Interactive psychoeducational workshops	Once a week	Social pedagogue	Based on cognitive and behavioural therapy in the form of learning new patterns of behaviour and thinking, the role of psychoeducation in the treatment protocol is to stimulate the process of change, raise awareness of one’s condition, provide information about addiction, and give specific recommendations for stable abstinence.
Cognitive-behavioural therapy	Once a week	Psychologist	The goal of cognitive behavioural therapy is to help patients become aware of their irrational thoughts and beliefs about gambling that have contributed to the development and maintenance of addiction and to strengthen their skills by learning specific techniques and strategies (such as coping strategies, cognitive restructuring techniques, and functional behaviour analysis).
Multifamily therapy	Once a week	Psychiatrist, social pedagogue	Considering the principles of systemic family therapy, elements of structural and strategic therapy, narrative therapy and solution-orientated therapy are used in this component of the treatment. By including the family in the treatment protocol, a better insight is gained into the patient’s functioning and the extent of gambling harm. The family is supported in recovery and their positive influence (as a significant other) on the treatment of gambling addiction is strengthened.
Support groups	Once a week	Day hospital patients (stable abstainers)	This part of the treatment is based on self-help groups, which have a long tradition in addiction treatment, and is also a form of aftercare, as it is open to all patients who have completed treatment. This component aims to encourage patients to take an active role in their treatment, to give them hope for recovery, and to enable them to learn from stable abstinence in a safe environment.
Individual psychotherapy	Continuous/as needed	Psychiatrist	All patients are offered supportive psychotherapy, while more extensive psychotherapeutic procedures are carried out if necessary to provide help and support in crisis situations and to maintain the patient’s motivation to continue abstinence.
Psychopharmaco-therapy	Continuous/as needed	Psychiatrist	The primary purpose of psychopharmacotherapy is to reduce the severity of gambling symptoms and to treat psychiatric comorbidities associated with gambling disorder, such as anxiety and depression (which are usually reactive in nature but precede the development of addiction in some patients and are a risk factor for relapse if left untreated).

Before starting with the treatment programme, a psychiatrist conducts an initial assessment to evaluate the patient’s motivation and readiness for treatment, as well as to identify any potential exclusion criteria. These may include circumstances or conditions that could impede the treatment process, such as significant impairments in intellectual functioning, complete illiteracy, or acute mental illness and/or personality disorders that would interfere with the process and/or outcomes. Through all components of the programme, opportunities to change behaviour, attitudes, and values are provided, while the emphasis is put on motivating the patient to actively participate with the final aim of changing his/her behaviour and, consequently, building better relationships and improving their quality of life.

From the opening of the daily clinic in 2015 to the end of 2022, a total of 870 patients have completed treatment, with a continuous increase in the number of those seeking treatment (e.g., 34 patients treated in 2015 compared to 220 in 2022). The constant growth in patients clearly demonstrates the need for such specialised gambling addiction treatment programme within the healthcare system as well as the need to scientifically evaluate it and modify accordingly.

### Evaluation and procedure

In 2017, a collaboration was established between the University Clinic “St John” and the University of Zagreb Faculty of Education and Rehabilitation Sciences to initiate a systematic scientific evaluation project for this gambling treatment. A design with two measurements sessions (pre-test and post-test) was included in the treatment protocol. A pre-test (T1) is administered at the beginning of the treatment, while the post-test (T2) is administered at the end of the treatment, i.e., prior to patients’ discharge from the clinic. Furthermore, this study follows a per protocol design, as only patients who completed the full treatment programme were included in the analysis. In terms of content, the questionnaire focuses on the constructs or areas of psychosocial functioning that are targeted by the treatment and in which changes are expected. Since the questionnaire is very comprehensive, its’ administration to patients is structured into four distinct parts spread across 4 days. Given the clinical setting, participant evaluations are non-anonymous, yet voluntary participation is ensured with informed consent obtained from all participants. A control group was not included in this study due to the fact that all individuals identified as gambling addicts are receiving treatment, and alternative treatment modalities are not available within the clinic. The study was approved by the Ethics Committee of the University of Zagreb Faculty of Education and Rehabilitation Sciences.

### Participants

Study comprised a clinical sample of *N* = 209 patients who have completed the outpatient treatment in the Daily Clinic for Gambling Addiction (Psychiatric Hospital “St. John,” Zagreb, Croatia). They represent the whole treatment-seeking population in this clinic in the period from December 2017 to December 2021. The sample ranged in age from 18 to 59 years, with a mean age of 33.54 (SD = 9.46); 92.8% were men. More than half were either married (43.5%) or in a relationship (13.9%) while 41.6% had children. The majority of the sample (77.4%) reported high school as their highest level of formal education and 66% were employed full time.

### Measures

An evaluation questionnaire consists of a comprehensive battery of the following instruments:

**General socio-demographic variables**. As part of these questions, patients provided information on gender, age, highest level of education, relationship status, number of children, employment status.

Additionally, participants were asked to identify their preferred and most frequently played game of chance among seven options (e.g., roulette, sports betting, etc.). To evaluate substance use (alcohol, cigarettes, and marijuana/hashish), participants were asked to indicate frequency of consumption over the past 3 months using a six-point scale (0-never; 5-everyday).

**Current treatment/motivational status**. To assess their treatment and motivation status, participants were asked to choose the statement that best described their current situation from seven options (example: “I have no intention to change my gambling behaviour.”; “I have completely stopped gambling more than 6 months ago.”). These statements were based on the stages of change as defined by the Prochaska and DiClemente model of change (Prochaska and DiClemente, 1983, 1992, 1986; DiClemente et al., 2004).

**Problem Gambling Severity Index** (**PGSI**; Ferris and Wynne, 2001) was used to assess gambling-related problems/symptoms. It is a nine-item scale where participants rate the extent to which each item applies to them on a four-point scale (0-never; 3-almost always) (example: “When you gambled, did you go back another day to try to win back the money you lost?”). A higher total score across all questions indicates a greater severity of problems. The Cronbach’s alpha coefficient in this study demonstrated good internal consistency at both measurement points (*α*T1 = 0.808, *α*T2 = 0.885).

To assess attitudes towards gambling attitudes, two measures were administered. **Gambling Attitudes Scale—GAS** (Jelić et al., 2013) is a 23-item scale where participants indicate the extent to which they agree with a particular statement on a five-point Likert scale (1-strongly disagree, 5-strongly agree) (example: “Gambling is a harmless adventure.”). The scale is unidimensional, and a higher overall average score indicates a more positive attitude towards gambling. At both time points, this scale has an acceptable internal reliability (*α*T1 = 0.738, *α*T2 = 0.607). Additionally, attitudes were also assessed using an Attitudes Towards Gambling Scale—ATGS-8 (Orford et al., 2009)—a widely used instrument in international research. Similarly, participants are asked to indicate how much they agree or disagree with each of the eight statements (example: “Gambling livens up life”) using a five-point Likert scale (1-strongly disagree; 5-strongly agree). The ATGS-8 total score is derived from the sum of all items, with a higher score indicating a more positive attitude towards gambling. The scale showed acceptable internal consistency at both measurement points (*α*T1 = 0.601, *α*T2 = 0.596).

**Coping Inventory for Stressful Situations—CISS** (Endler and Parker, 1990) was employed to evaluate various coping strategies. It comprises 48 items distributed across three subscales (16 items each) that address different dimensions of coping with stress: (1) emotion-oriented coping (e.g., “Blame myself for not knowing what to do.”), (2) task/problem-oriented coping (e.g., “Think about how I have solved similar problems.”), and (3) avoidance-oriented coping (e.g., “Go out for a snack or meal.”). Participants rated the frequency of engaging in each activity or behaviour on a five-point scale (1 = not at all; 5 = yes, very much) when faced with stressful situations. Higher scores on each subscale indicate a greater tendency to use those coping strategies. In the sample of participants in this study, the Cronbach’s alphas for the individual subscales are as follows: Emotion-oriented coping: *α*T1 = 0.834, *α*T2 = 0.871; Task/problem-oriented coping: *α*T1 = 0.886, *α*T2 = 0.878; Avoidance-oriented coping: *α*T1 = 0.752, *α*T2 = 0.795, confirming good psychometric properties.

**The Gambling Beliefs Scale—short version** (Ricijaš et al., 2011) assesses cognitive distortions related to gambling. It comprises two subscales: (1) Superstition and misinterpretation of chances and probability (6 items; e.g., “Lucky charms (e.g., clothing, talismans, lucky charms, etc.) increase the likelihood of winning in gambling.”) and (2) Illusion of control (7 items; e.g., “Over time, gambling can result in more gains than losses.”). Participants rate their agreement with each item on a five-point scale ranging from 1 (strongly disagree) to 5 (strongly agree). The scale generates separate scores for each subscale, with higher average overall scores indicating greater cognitive distortions related to gambling. In this study’s participant sample, Cronbach’s alphas indicate satisfactory internal consistency for both subscales at both measurement points (Superstition and misinterpretation of chances and probability: *α*T1 = 0.755, *α*T2 = 0.824; Illusion of control: *α*T1 = 0.759, *α*T2 = 0.610).

**Problem Solving and Refusal Skills Scale** (Huic et al., 2017) comprises two subscales: (1) Problem-solving skills (5 items; e.g., “I know how to assess whether I have successfully solved my problem.”) and (2) Refusal (peer pressure) skills (5 items; e.g., “I know how to resist the pressure that others put on me.”). Participants rated their responses on a five-point scale (0 – never; 4 – almost always) based on their typical responses to various situations. Higher average scores on each subscale indicate better problem-solving and refusal skills. However, the internal consistency of both subscales was found to be poor (Problem-solving skills: *α*T1 = 0.858, *α*T2 = 0.487; Refusal skills: *α*T1 = 0.590, *α*T2 = 0.431). Therefore, caution is advised when interpreting these results, underscoring the necessity for using a more suitable instrument to measure these constructs.

**Depression, Anxiety, Stress Scale—DASS-21** (Lovibond and Lovibond, 1995) is widely used to assess depression (example: “I could not seem to experience any positive feeling at all.”), anxiety (example: “I was worried about situations in which I might panic and make a fool of myself.”) and stress (example: “I was intolerant of anything that kept me from getting on with what I was doing.”). The participants answer each of the 21 statements (on a four-point scale: 0-never, 3-almost always) and thus indicate how often they experienced the condition described in the item during the period analysed. In this study, very good metric properties were confirmed on all subscales at both measurement times (Depression: *α*T1 = 0.917, *α*T2 = 0.851; Anxiety: *α*T1 = 0.853, *α*T2 = 0.794; Stress: *α*T1 = 0.904, *α*T2 = 0.875).

**Generalised Self-Efficacy Scale—GSE** (Schwarzer and Jerusalem, 1995) consists of 10 items and measures one’s general sense of perceived self-efficacy and belief that one can master new or difficult tasks or cope with adversity in various areas of human functioning (example: “I can solve my problems if I invest the necessary effort.”). Responses for each item ranged from 1 (strongly disagree) to 5 (strongly agree), and the composite score is calculated as the average score for all items, with a higher score indicating higher perceived generalised self-efficacy. In this study, the scale had high internal consistency at both time points (*α*T1 = 0.883, *α*T2 = 0.904).

**Multidimensional Scale of Perceived Social Support** (Zimet et al., 1988) was administered to measure two dimensions of perceived social support: family support (4 items; example: “My family really tries to help me.”) and significant other support (4 items; example: “There is a special person in my life who cares about my feelings.”). Participants rated their agreement with each statement on a seven-point scale ranging from 1 (strongly disagree) to 7 (strongly agree). Higher scores on each subscale indicate greater perceived social support from the particular source. The scale demonstrated strong internal consistency for both subscales at both measurement points (Family support: *α*T1 = 0.909, *α*T2 = 0.933; Significant other support: *α*T1 = 0.932, *α*T2 = 0.958) in this study.

### Data analysis

The first step was to analyse the skewness and kurtosis in pre-test and post-test for all variables whose changes were to be assessed for the entire sample (Table 2). This analysis followed guidelines proposed by Kim (2013), who recommend that for sample sizes between 50 and 300, the *z*-value should fall within the range of −3.29 to +3.29 for a distribution to be considered normal.

**Table 2:** Skewness, kurtosis and Cronbach’s alpha reliability for all variables (*N* = 209).

	All sample (*N* = 209)		
	Skew.	Kurt.	*α*
Total PGSI (T1)	−0.53	−0.28	**0.808**
Total PGSI (T2)	9.28	5.77	**0.885**
GAS_ attitudes (T1)	4.64	2.71	**0.738**
GAS_ attitudes (T2)	7.79	8.11	0.607
ATGS_attitudes (T1)	3.94	0.00	0.601
ATGS_attitudes (T2)	5.24	0.99	0.596
CISS_task (T1)	−3.86	1.29	**0.886**
**CISS_emotions (T1)**	**0.20**	**0.36**	**0.834**
**CISS_avoidant (T1)**	**0.88**	**0.42**	**0.752**
CISS_task (T2)	−2.82	4.72	**0.878**
**CISS_emotions (T2)**	**0.76**	**−1.06**	**0.871**
**CISS_avoidant (T2)**	**0.51**	**2.04**	**0.795**
CD_superstition_probability (T1)	12.77	18.91	**0.755**
CD_illusion of control (T1)	7.62	3.78	**0.759**
CD_superstition_probability (T2)	50.57	272.98	**0.824**
CD_illusion of control (T2)	17.19	29.57	**0.610**
DASS_depression (T1)	0.75	−2.72	**0.917**
DASS_anxiety (T1)	4.01	−0.40	**0.853**
DASS_stress (T1)	0.71	−2.21	**0.904**
DASS_depression (T2)	7.95	7.49	**0.851**
DASS_anxiety (T2)	11.57	15.58	**0.794**
DASS_stress (T2)	6.27	6.26	**0.875**
GSE_general self-efficacy (T1)	−6.01	5.14	**0.883**
GSE_general self-efficacy (T2)	−1.99	3.11	**0.904**
Social Support_significant other (T1)	−9.86	6.94	**0.932**
Social Support _family (T1)	−10.95	10.56	**0.909**
Social Support _significant other (T2)	−12.53	13.38	**0.958**
Social Support _family (T2)	−11.95	13.83	**0.933**
Cigarettes (T1)	−6.02	−2.65	n.a.
**Alcohol (T1)**	**1.23**	**−2.07**	n.a.
Marihuana (T1)	18.51	28.52	n.a.
Cigarettes (T2)	−6.04	−2.69	n.a.
**Alcohol (T2)**	**2.19**	**−1.87**	n.a.
Marihuana (T2)	25.91	67.04	n.a.
Problem solving (T1)	−2.10	0.20	**0.858**
**Refusal (T1)**	**−1.53**	**2.54**	0.591
Problem solving (T2)	20.84	103.08	0.487
**Refusal (T2)**	**−0.21**	**0.79**	0.431

Skew., Skewness; Kurt., Kurtosis; *α*, Cronbach’s Alpha; PGSI, Problem Gambling Severity Index; GAS, Gambling Attitudes Scale; ATGS, Attitudes Towards Gambling Scale; CISS, Coping Inventory for Stressful Situations; CD, cognitive distortion; DASS, Depression, Anxiety, Stress Scale; GSE, Generalised Self-Efficacy Scale. Bold variables suitable for parametric analysis.

The results indicated that parametric analysis was suitable only for two dimensions of the Coping Inventory for Stressful Situations (CISS): emotion-oriented coping and avoidant-oriented coping, as well as for refusal skill and alcohol consumption. Therefore, a paired-samples t-test with effect size was performed for these variables, while a non-parametric Wilcoxon rank test was used for all other variables to compare differences between pre- and post-test measurements.

Due to the extensive number of statistical comparisons (totalling 19), and to mitigate the risk of inference errors (Petz et al., 2012; Armstrong, 2014), we applied the Bonferroni correction. This adjustment aimed to establish a more stringent criterion for statistical significance. Consequently, the Bonferroni correction in this study set the threshold for statistical significance at *p* < 0.00263.

## Results

In the introduction, it was highlighted that gambling and associated issues constitute a significant public health concern in Croatia, facilitated by widespread accessibility to various forms of gambling. Given the varying addictive potentials of different games of chance (James et al., 2016; Allami et al., 2021; Flayelle et al., 2023; Gooding and Williams, 2024; Russell et al., 2023), our study aimed to identify which types are most appealing to individuals seeking treatment for gambling problems, i.e., which game of chance is their favourite and dominant. The results showed that nearly half of the participants (49.3%) preferred electronic gaming machines (EGMs), approximately one-third (32.1%) favoured sports betting, and 15.8% preferred roulette. These preferences indicate that the favoured activities are those typically associated with a higher addictive potential, contributing to gambling-related problems and the development of gambling addiction.

Given that motivation for treatment and motivation for behavioural change are extremely important factors when it comes to the effectiveness of the intervention, self-assessment of current motivation is also an integral part of the initial questionnaire (Table 3). As can be seen from the results presented, a third of the sample (30.1%) are seriously considering reducing or quitting gambling in the following 6 months. A similar proportion (29.1%) plan to reduce or quit gambling in the following month, while 18.7% have already started to reduce gambling in the past 6 months. A non-negligible proportion (13.9%) have stopped gambling completely within the last 6 months.

**Table 3:** Current treatment/motivational status (*N* = 209).

Statement	%
1. I have no intention to change my gambling behaviour.	0.0
2. I am seriously thinking of reducing or quitting gambling in the following 6 months.	30.1
3. I am planning to reduce or quit gambling in the following month.	29.1
4. I have started to reduce gambling in the past 6 months.	18.7
5. I have started to reduce gambling more than 6 months ago.	4.8
6. I have completely stopped gambling within the past 6 months.	13.9
7. I have completely stopped gambling more than 6 months ago.	2.9

The short-term intervention effects were tested performing a paired-sample t-test and a non-parametric Wilcoxon rank test, depending on the normality of the distribution of the results (Table 4). To understand the magnitude of the potential differences, the effect sizes were calculated (Cohen’s *d* for the paired samples t-test and r for the Wilcoxon rank test), with values above 0.2 being considered small effects, above 0.5 as medium effects and those exceeding 0.8 as large.

**Table 4:** Mean scores compared with paired-samples t-test/Wilcoxon rank test at pre- and post-test (short-term effectiveness) (*N* = 209).

	Variables (theoretical range)	Pre-test (T1)		Post-test (T2)		*Z*/*t*	*r*/Cohen’s *d*
		*M*	SD	*M*	SD		
Gambling problems	**PGSI TOTAL (0–27)**	15.84	4.90	4.15	5.37	−12.088*	**0.84**
Attitudes	**GAS (CRO)** (1–5)	1.70	0.39	1.39	0.27	−9.682*	**0.67**
	ATGS (8–40)	13.38	3.97	11.84	3.72	−5.577*	0.39
Cognitive distortions	**SUPERSTITION/PROBABILITY** (1–5)	1.34	0.48	1.05	0.20	−8.793*	**0.61**
	**THE ILLUSION OF CONTROL** (1–5)	1.54	0.60	1.12	0.26	−9.034*	**0.62**
Psychoactive substance use	CIGARETTES (0–5)	3.62	2.14	3.62	2.16	−0.379 (n.s.)	/
	ALCOHOL (0–5)	1.62	1.23	1.36	1.08	3.509*	0.24
	MARIHUANA/HASHISH (0–5)	0.36	0.97	0.15	0.51	−3.694*	0.26
Mental health	**DEPRESSION** (0–42)	9.88	5.67	3.64	3.50	−11.292*	**0.78**
	**ANXIETY** (0–42)	6.86	4.79	2.60	3.03	−10.327*	**0.71**
	**STRESS** (0–42)	10.65	5.13	5.65	3.98	−10.589*	**0.73**
Socio-emotional skills	**GENERAL SELF-EFFICACY** (10–50)	34.37	6.46	39.45	5.88	−8.882*	**0.61**
	**PROBLEM SOLVING** (0–4)	3.19	0.74	3.96	0.85	−10.614*	**0.73**
	REFUSAL (0–4)	3.21	0.60	3.52	0.49	−6.922*	0.48
	**COPING—task oriented** (16–80)	49.83	9.54	61.14	7.94	−11.413*	**0.79**
	**COPING—emotion oriented** (16–80)	50.75	9.04	40.25	9.68	15.141*	**1.06**
	**COPING—avoidant social** (16–80)	42.49	8.39	44.91	8.20	−4.084*	0.28
Social support	SIGNIFICANT OTHER (1–7)	5.94	1.39	6.23	1.25	−4.135*	0.29
	FAMILY (1–7)	6.07	1.21	6.28	1.06	−2.560*	0.18

n.s., non-significant; *p < 0.002; PGSI, Problem Gambling Severity Index; GAS, Gambling Attitudes Scale; ATGS, Attitudes Towards Gambling Scale. Bold medium to large effect sizes.

The findings indicate statistically significant differences across all variables except cigarette consumption (which is not a focus of the treatment, but still measured variable in the context of addictive behaviours). Regarding the effect sizes of these differences, they predominantly reflect medium to large magnitudes. Notably, the most substantial effects (exceeding 0.8) were observed for gambling-related problems, as measured by the Problem Gambling Severity Index (PGSI). Initially, the mean PGSI score was 15.84, decreasing to 4.15 post-treatment.

Furthermore, the effects are also high for emotion-oriented coping strategies, while medium (approaching high) effects were observed for mental health variables; specifically, patients reported fewer symptoms of depression, anxiety, and stress (measured by DASS-21) at the end of the treatment. Medium effect sizes were also noted for general self-efficacy and problem-solving skills, as well as for shifts towards more negative attitudes regarding gambling. For cognitive distortions, a construct that is extremely important in relation to gambling and gambling-related problems, we also found medium effect sizes, indicating reductions in superstitions, erroneous probability beliefs and illusion of control following treatment. Conversely, minimal or negligible effects were observed for psychoactive substance use (alcohol and marijuana), refusal skills, and social support from significant others and family members.

Overall, an important feature of these results is the consistent direction of effects, characterized by reduced gambling problems, more negative attitudes, lower levels of cognitive distortions, better socio-emotional skills and increased self-efficacy, demonstrating the effectiveness of this treatment intervention.

## Discussion

Short-term effectiveness evaluation of the gambling treatment programme in the Daily Clinic for Gambling Addiction suggests that this treatment achieves its’ purpose. Specifically, the findings demonstrate significant reductions in gambling-related problems, i.e., symptoms of gambling addiction, cognitive distortions and mental health problems (depression, anxiety, and stress). It also contributes to more negative attitudes towards gambling and improves overall self-efficacy, emotion-oriented coping skills and problem-solving skills. However, the programme showed limited impact on certain factors such as the ability to handle pressure (refusal skills), support from significant others, and the use of psychoactive substances.

Regarding the latter, there were minor effects observed on alcohol and marijuana/hashish consumption, while no significant difference was found in cigarette use between the two assessment points. It is noteworthy that the average alcohol and marijuana use among participants was generally low, regardless of the treatment, which also explains the lack of significant differences observed between the two assessment points. Furthermore, considering the characteristics of cigarette use and the fact that it does not affect the individual’s psychosocial functioning in a psychosocial sense and is not significant as a risk factor for (re)engaging in gambling activities, the obtained result is expected and logical.

Regarding social support from significant others and family members, the effects observed are statistically significant but relatively modest (0.29 and 0.18, respectively). However, when comparing mean scores before and after treatment, these outcomes are logical. Initially, the average scores for support from significant others and family members were notably high (5.94 and 6.07, respectively, on a theoretical maximum of 7). This result is important for two possible reasons. Firstly, it suggests that social support plays a crucial role in treatment engagement; individuals with strong support systems are more likely to initiate and adhere to treatment, a trend supported by previous research (Dowling et al., 2006; Jiménez-Murcia et al., 2007; Hing et al., 2014; Kourgiantakis et al., 2018). Furthermore, we can note that in light of this finding, the other effects of the study should be considered, i.e., that these results align with broader findings in the field of addiction and risk behaviour treatments, underscoring the enhanced effectiveness of interventions when accompanied by social support from significant others (Gomes, 2017; Gomes and Pascual-Leone, 2014).

Regarding cognitive variables such as cognitive distortions and attitudes towards gambling, they consistently emerge as critical predictors of gambling-related problems and as a fundamental component of gambling addiction treatment. Specifically, cognitive distortions play a central role in all pathways leading to problem gambling development according to the prominent integrative theory, the Pathways model (Blaszczynski and Nower, 2002). This correlation is unsurprising, as research consistently shows that irrational beliefs related to gambling are significantly more common among individuals who have developed gambling addictions (Steenbergh et al., 2002; May et al., 2005; Cocker and Winstanley, 2015; Armstrong et al., 2020; Choi, 2021; Choi and Kim, 2021). As far as attitudes are concerned, the studies are divergent in findings concerning their relation to problem gambling (Hellumbråten Kristensen et al., 2022). However, they are undoubtedly a relevant cognitive variable, and the numerous research findings in favour of more positive attitudes in problem gamblers should not be ignored (Chiu and Storm, 2009; Orford et al., 2009, 2010; Canale et al., 2016; Zhou et al., 2018; Andrà et al., 2021). Considering all of the above, as well as the fact that all available treatment models emphasise the importance of focusing on cognition (Sylvain et al., 1997; Ladouceur et al., 2003; Petry, 2005; Dowling et al., 2006; Jiménez-Murcia et al., 2007; Rizeanu, 2015; Garcia-Caballero et al., 2018) through a cognitive-behavioural therapeutic approach and cognitive restructuring, addressing cognitive variables is one of the integral elements of this treatment. Our findings also confirm that cognition is a construct that can be effectively influenced in treatment interventions. More specifically, the results of this study show a significant decrease in the illusion of control, superstition and incorrect understanding of probability (with medium effect sizes). Furthermore, there was a notable shift towards more negative (critical) attitudes towards gambling post-treatment, as measured by the ATGS-8 (Orford et al., 2009) scale (small effect) and the Gambling Attitude Scale (GAS, Jelić et al., 2013) (medium effect).

While specific cognition is an important mechanism underlying risky behaviours, gambling addiction included, one of the fundamental postulates of treatment interventions is that effective programmes are those that sufficiently address socio-emotional skills (Jiménez-Murcia et al., 2007; Petry et al., 2007; Ledgerwood et al., 2013; Gomes and Pascual-Leone, 2014). Therefore, the evaluation of this treatment intervention also measured effects on coping, problem-solving, refusal skills, and general self-efficacy. Given that it is challenging to determine the treatment effects on social and emotional skills in a short-term period, with a paper-pencil evaluation design, it is particularly noteworthy that significant effects were found on all variables measured and in the desired direction. Specifically, this intervention improved the patients’ refusal skills, problem-solving skills, coping with stress and general self-efficacy. The effects were smallest when it comes to the refusal skill and avoidance-orientated coping, medium for problem-solving and general self-efficacy, and largest for task-orientated (0.79) and emotion-orientated coping (1.06). However, these findings should be interpreted carefully, particularly in relation to problem-solving and refusal skills, due to the scale’s noted low internal consistency. Nevertheless, these results are not unexpected given the methodological complexities involved in assessing socio-emotional competencies and their enhancement through psychosocial interventions, which certainly is an important implication for subsequent evaluation designs.

Gambling addiction often co-occurs with other mental health problems, and the mechanisms linking them are complex. These comorbidities can be reactive; other mental health problems may emerge as a direct result of gambling and its detrimental impacts. Conversely, emotional and psychological disorders can also predispose individuals to develop gambling-related issues, with gambling sometimes serving as a coping mechanism to manage stress or adverse mental states (Blaszczynski and Nower, 2002; Griffiths, 2005). Therefore, addressing psychiatric comorbidities through both pharmacotherapy and psychotherapy is crucial in treatment protocols. In this evaluation study, we investigated whether treatment had an effect on symptoms of depression, anxiety and stress as measured by the DASS-21 (Lovibond and Lovibond, 1995) instrument, and positive effects with medium to large effect differences were found. Considering these findings alongside the enhancement of coping skills demonstrated earlier, this holds particular significance for preventing gambling relapse.

Finally, but not least, the greatest impact of the differences (0.84) was found in the level of adverse psychosocial consequences of gambling, i.e., the total score of the Problem Gambling Severity Index (PGSI) instrument. Specifically, the average score on this measure decreased from the initial 15.84 to 4.15, indicating that the treatment contributed significantly to reducing the harmful consequences of gambling, which is one of its main objectives. Unfortunately, we lack data regarding the treatment’s effectiveness in achieving sustained abstinence from gambling, which is another primary goal of the intervention. This leads us to the limitations of this study, which are important to acknowledge and address in future research within the field.

## Study limitations

Our study has several limitations that need to be considered, the main one being related to its’ short-term design, i.e., the fact that there is insufficient evidence of its long-term effectiveness, crucial for understanding the enduring impact of the treatment, particularly concerning behavioural changes that may require time and are challenging to capture with traditional paper-pencil methods.

In the context of methodological shortcomings, it is important to also point out the challenges of self-reporting, which may be susceptible to biases such as the memory effect and the tendency to provide socially desirable responses (Razavi, 2001; van de Mortel, 2008; Uttl and Kibreab, 2011). Furthermore, although the results clearly indicate significant effects on socio-emotional skills and cognitive distortions, these are generally difficult to measure. This is supported by the fact that, for example, average values for cognitive distortions were low even at the initial assessment, which may be attributed to measurement challenges, as they typically manifest as automatic thoughts during gambling situations, making accurate assessment in a neutral setting using the paper-pencil method difficult. In terms of socio-emotional skills, it would also be preferable to include other assessment methods, i.e., those that allow patients to demonstrate their ability to use these skills in addition to, of course, assessing them in a follow-up study.

Recognising that the length of the instrument could also be a challenge and a potential shortcoming, completion of the questionnaire was split into four parts over 4 days, which proved to be a good approach.

Furthermore, it would be valuable to analyse predictors of varying degrees of treatment success among those who completed the intervention. Understanding the role of personality traits, cognitive factors, behaviours, social support, environmental influences, and the potential contribution of pharmacotherapy can provide further insight into the effectiveness of this multifaceted intervention. In this context, given that this study included only patients who successfully completed the entire treatment in accordance with the defined protocol, future research should explore the characteristics of individuals who discontinued treatment, the underlying reasons for their attrition, and the potential barriers to treatment adherence and completion.

Additionally, it is essential to acknowledge that the sample predominantly consists of men, which is not surprising given that gambling and problem gambling are primarily male-dominated phenomena (Glavak Tkalic et al., 2017; Stoltenberg et al., 2008; Welte et al., 2004). However, due to this composition of treatment-seeking patients, the conclusions drawn about treatment effectiveness are primarily applicable to the male population. Therefore, future studies should examine potential gender-specific differences both in terms of treatment needs and intervention outcomes.

Considering these findings collectively, along with their potential limitations, they underscore the necessity for ongoing research in this field. This includes exploring the long-term effectiveness of the treatment, examining predictors of treatment success, and focusing on the involvement of significant others in the process.

## Conclusion

Despite its limitations, this study provides evidence that the gambling treatment programme at the Daily Clinic for Gambling Addiction is effective in reducing the adverse consequences of gambling and improving overall psychosocial functioning. The data suggest that treatment yields significant improvements in gambling-related consequences, attitudes, cognitive distortions, comorbid mental health problems, problem-solving skills, and general self-efficacy, while the effects on refusal skills and psychoactive substance use are small or negligible.

The development of this programme is based on research findings on gambling behaviour and the predictors of problem gambling, on the principles of evidence-based interventions and on established treatment methods. It is therefore a good example of a comprehensive and multimodal approach that has the potential to effectively treat this complex disorder.

Furthermore, given the lack of evaluation studies on the treatment of gambling addiction, this study represents an important contribution to the existing pool of knowledge. The findings underscore the need for further research on the short-and long-term effectiveness of interventions with the aim of overcoming methodological and implementation-related limitations and reaching a consensus on effective treatment modalities for gambling addiction.

## Data Availability

The raw data supporting the conclusions of this article will be made available by the authors without undue reservation.

## References

[ref1] Abbott M. Bellringer M. Vandal A. C. Hodgins D. C. Battersby M. Rodda S. N. (2017). Effectiveness of problem gambling interventions in a service setting: a protocol for a pragmatic randomised controlled clinical trial. BMJ Open 7:e013490. doi: 10.1136/bmjopen-2016-013490, PMID: 28255094 PMC5353265

[ref2] Allami Y. Hodgins D. C. Young M. Brunelle N. Currie S. Dufour M.. (2021). A meta-analysis of problem gambling risk factors in the general adult population. Addiction 116, 2968–2977. doi: 10.1111/add.15449, PMID: 33620735 PMC8518930

[ref3] Russell A. M. T. Browne M. Hing N. Rockloff M. Newall P. Dowling N. A.. (2023). Electronic gaming machine accessibility and gambling problems: a natural policy experiment. J. Behav. Addict. 12, 721–732. doi: 10.1556/2006.2023.00044, PMID: 37594879 PMC10562817

[ref4] Andrà C. Priolo G. Merlin F. Chiavarino C. (2021). Differences in perceived and experienced stigma between problematic gamblers and non-gamblers in a general population survey. J. Gambl. Stud. 38, 333–351. doi: 10.1007/s10899-021-10048-9, PMID: 34216324 PMC9119868

[ref5] Armstrong R. A. (2014). When to use the Bonferroni correction. Ophthalmic Physiol. Opt. 34, 502–508. doi: 10.1111/opo.12131, PMID: 24697967

[ref6] Armstrong T. Rockloff M. Browne M. (2020). Gamble with your head and not your heart: a conceptual model for how thinking-style promotes irrational gambling beliefs. J. Gambl. Stud. 36, 183–206. doi: 10.1007/s10899-019-09927-z, PMID: 31912382

[ref7] Blank L. Baxter S. Woods H. B. Goyder E. (2021). Interventions to reduce the public health burden of gambling-related harms: a mapping review. Lancet Public Health 6, e50–e63. doi: 10.1016/S2468-2667(20)30230-9, PMID: 33417847

[ref8] Blaszczynski A. Nower L. (2002). A pathways model of problem and pathological gambling. Addiction 97, 487–499. doi: 10.1046/j.1360-0443.2002.00015.x, PMID: 12033650

[ref9] Bodor D. (2018). Usporedba psihosocijalnoga funkcioniranja osoba koje se liječe zbog ovisnosti o kockanju i alkoholu. [PhD thesis]. Available online at: https://dr.nsk.hr/islandora/object/sfzg%3A448 (Accessed November 27, 2024).

[ref10] Bodor D. Ricijaš N. Filipčić I. (2021). Treatment of gambling disorder: review of evidence-based aspects for best practice. Curr. Opin. Psychiatry 34, 508–513. doi: 10.1097/yco.0000000000000728, PMID: 34282103

[ref11] Bodor D. Ricijas N. Zoricic Z. Dodig Hundric D. Filipčić I. (2018). Prevalence of pathological gambling among alcohol addicts in outpatient treatment in the City of Zagreb: a cross-sectional study. Psychiatr. Danub. 30, 348–355. doi: 10.24869/psyd.2018.348, PMID: 30267528

[ref12] Booth N. Dowling N. A. Landon J. Lubman D. I. Merkouris S. S. Rodda S. N. (2021). Affected others responsivity to gambling harm: an international taxonomy of consumer-derived behaviour change techniques. J. Clin. Med. 10:583. doi: 10.3390/jcm10040583, PMID: 33557212 PMC7913932

[ref13] Bruneau M. Grall-Bronnec M. Vénisse J.-L. Romo L. Valleur M. Magalon D.. (2016). Gambling transitions among adult gamblers: a multi-state model using a Markovian approach applied to the JEU cohort. Addict. Behav. 57, 13–20. doi: 10.1016/j.addbeh.2016.01.010, PMID: 26827154

[ref14] Calado F. Griffiths M. D. (2016). Problem gambling worldwide: an update and systematic review of empirical research (2000–2015). J. Behav. Addict. 5, 592–613. doi: 10.1556/2006.5.2016.073, PMID: 27784180 PMC5370365

[ref9001] Calado F. Alexandre J. Griffiths M. D. (2017). Prevalence of Adolescent Gambling: A systematic review of Recent Research. Journal of Gambling Studies, 33, pp.397–424. doi: 10.1007/s10899-016-9627-5 27372832 PMC5445143

[ref15] Canale N. Vieno A. Pastore M. Ghisi M. Griffiths M. D. (2016). Validation of the 8-item attitudes towards gambling scale (ATGS-8) in a British population survey. Addict. Behav. 54, 70–74. doi: 10.1016/j.addbeh.2015.12.009, PMID: 26722993

[ref16] Carlbring P. Jonsson J. Josephson H. Forsberg L. (2010). Motivational interviewing versus cognitive behavioral group therapy in the treatment of problem and pathological gambling: a randomized controlled trial. Cogn. Behav. Ther. 39, 92–103. doi: 10.1080/16506070903190245, PMID: 19967577 PMC2882846

[ref17] Carroll K. M. Ball S. A. Nich C. Martino S. Frankforter T. L. Farentinos C.. (2006). Motivational interviewing to improve treatment engagement and outcome in individuals seeking treatment for substance abuse: a multisite effectiveness study. Drug Alcohol Depend. 81, 301–312. doi: 10.1016/j.drugalcdep.2005.08.002, PMID: 16169159 PMC2386852

[ref18] Chiu J. Storm L. (2009). Personality, perceived luck and gambling attitudes as predictors of gambling involvement. J. Gambl. Stud. 26, 205–227. doi: 10.1007/s10899-009-9160-x 19943093

[ref19] Choi J. (2021). Effects of irrational beliefs, impulsivity, and happiness on problem gambling: focused on Korean and Australian college students. J. Digit. Converg. 19, 641–648. doi: 10.14400/JDC.2021.19.12.641

[ref20] Choi J. Kim K. (2021). The relationship between impulsiveness, self-esteem, irrational gambling belief and problem gambling moderating effects of gender. Int. J. Environ. Res. Public Health 18:5180. doi: 10.3390/ijerph18105180, PMID: 34068198 PMC8153021

[ref21] Cocker P. J. Winstanley C. A. (2015). Irrational beliefs, biases and gambling: exploring the role of animal models in elucidating vulnerabilities for the development of pathological gambling. Behav. Brain Res. 279, 259–273. doi: 10.1016/j.bbr.2014.10.043, PMID: 25446745

[ref22] Cowlishaw S. Merkouris S. Dowling N. Anderson C. Jackson A. Thomas S. (2012). Psychological therapies for pathological and problem gambling. Cochrane Database Syst. Rev. doi: 10.1002/14651858.cd008937.pub2, PMID: 23152266 PMC11955261

[ref23] Diaz-Sanahuja L. Campos D. Mira A. Castilla D. García-Palacios A. Bretón-López J. M. (2021). Efficacy of an internet-based psychological intervention for problem gambling and gambling disorder: study protocol for a randomized controlled trial. Internet Interv. 26:100466. doi: 10.1016/j.invent.2021.100466, PMID: 34646753 PMC8501496

[ref24] DiClemente C. C. Schlundt D. Gemmell L. (2004). Readiness and stages of change in addiction treatment. Am. J. Addict. 13, 103–119. doi: 10.1080/10550490490435777, PMID: 15204662

[ref9002] Derevensky J. Gupta R. Hardoon K. Dickson L. Deguire A. E. (2003). Youth gambling: Some social policy issues, in For Fun or Profit? The Controversies of the Expansion of Gambling, ed. Reith G. (New York: Prometheus Books), pp. 239–257.

[ref25] Dodig Hundric D. Mandic S. Ricijas N. (2021). Short-term effectiveness of the youth gambling prevention program ‘who really wins?’—results from the first national implementation. Int. J. Environ. Res. Public Health 18:10100. doi: 10.3390/ijerph181910100, PMID: 34639404 PMC8507822

[ref26] Dowling N. Smith D. Thomas T. (2006). Treatment of female pathological gambling: the efficacy of a cognitive-behavioural approach. J. Gambl. Stud. 22, 355–372. doi: 10.1007/s10899-006-9027-3, PMID: 16924426

[ref27] Dowling N. Smith D. Thomas T. (2007). A comparison of individual and group cognitive-behavioural treatment for female pathological gambling. Behav. Res. Ther. 45, 2192–2202. doi: 10.1016/j.brat.2006.11.003, PMID: 17196159

[ref28] Downs C. Woolrych R. (2010). Gambling and debt: the hidden impacts on family and work life. Community Work Fam. 13, 311–328. doi: 10.1080/13668803.2010.488096

[ref29] Echeburúa E. Fernández-Montalvo J. Báez C. (2000). Relapse prevention in the treatment of slot-machine pathological gambling: long-term outcome. Behav. Ther. 31, 351–364. doi: 10.1016/s0005-7894(00)80019-2

[ref30] Endler N. S. Parker J. D. (1990). Multidimensional assessment of coping: a critical evaluation. J. Pers. Soc. Psychol. 58, 844–854. doi: 10.1037/0022-3514.58.5.844, PMID: 2348372

[ref31] Ferris J. Wynne H. (2001). The Canadian problem gambling index: final report. Available online at: https://jogoremoto.pt/docs/extra/TECb6h.pdf (Accessed June 23rd, 2024).

[ref32] Flayelle M. Brevers D. King D. L. Maurage P. Perales J. C. Billieux J. (2023). A taxonomy of technology design features that promote potentially addictive online behaviours. Nat. Rev. Psychol. 2, 136–150. doi: 10.1038/s44159-023-00153-4

[ref33] Fong T. W. (2005). Types of psychotherapy for pathological gamblers. Psychiatry (Edgmont) 2:32. PMC3000184 21152147

[ref34] Garcia-Caballero A. Torrens-Lluch M. Ramírez-Gendrau I. Garrido G. Vallès V. Aragay N. (2018). Eficacia de la intervención Motivacional y la Terapia Cognitivo-conductual para el tratamiento del Juego Patológico. Adicciones 30, 219–224. doi: 10.20882/adicciones.965, PMID: 29353301

[ref35] Gavriel-Fried B. Delfabbro P. Ricijas N. Dodig Hundric D. Derevensky J. L. (2021). Cross-national comparisons of the prevalence of gambling, problem gambling in young people and the role of accessibility in higher risk gambling: a study of Australia, Canada, Croatia and Israel. Curr. Psychol. 42, 6990–7001. doi: 10.1007/s12144-021-02017-7, PMID: 40078838

[ref9003] Glavak Tkalic R. Miletic G. M. Sucic L. (2017). Igranje igara na srecu u hrvatskom drustvu. Zagreb: Institut drustvenih znanosti Ivo Pilar & Ured za suzbijanje zlouporabe droga Vlade Republike Hrvatske. Available at: https://www.pilar.hr/wp-content/uploads/2018/01/IGRANJE_IGARA_NA_SRECU_U_HRVATSKOM_DRUSTVU.pdf (Accessed 23 June 2024).

[ref36] Globan T. Rogić Dumančič L. Ricijaš N. Omazić M. A. (2021). Ekonomski i društveni troškovi ovisnosti o kockanju u Hrvatskoj – druga strana medalje. Ann. Soc. Work 28, 37–70. doi: 10.3935/ljsr.v28i1.413

[ref37] Gomes K. (2017). Facilitating effects of social support in the treatment of problem gambling. Scholarship at UWindsor. Available online at: https://scholar.uwindsor.ca/etd/7030?utm_source=scholar.uwindsor.ca%2Fetd%2F7030&utm_medium=PDF&utm_campaign=PDFCoverPages (Accessed November 27, 2024).

[ref38] Gomes K. Pascual-Leone A. (2014). A resource model of change: client factors that influence problem gambling treatment outcomes. J. Gambl. Stud. 31, 1651–1669. doi: 10.1007/s10899-014-9493-y, PMID: 25112220

[ref39] Gooding P. Tarrier N. (2009). A systematic review and meta-analysis of cognitive-behavioural interventions to reduce problem gambling: hedging our bets? Behav. Res. Ther. 47, 592–607. doi: 10.1016/j.brat.2009.04.002, PMID: 19446287

[ref40] Gooding N. B. Williams R. J. (2024). Are there riskier types of gambling? J. Gambl. Stud. 40, 555–569. doi: 10.1007/s10899-023-10231-0, PMID: 37355524

[ref41] Goodwin B. C. Browne M. Rockloff M. Rose J. (2017). A typical problem gambler affects six others. Int. Gambl. Stud. 17, 276–289. doi: 10.1080/14459795.2017.1331252

[ref42] Grande-Gosende A. López-Núñez C. García-Fernández G. Derevensky J. Fernández-Hermida J. R. (2019). Systematic review of preventive programs for reducing problem gambling behaviors among young adults. J. Gambl. Stud. 36, 1–22. doi: 10.1007/s10899-019-09866-9, PMID: 31168687

[ref43] Griffiths M. (2003). Problem gambling. Psychologist 16, 582–584.

[ref44] Griffiths M. (2005). A ‘components’ model of addiction within a biopsychosocial framework. J. Subst. Abus. 10, 191–197. doi: 10.1080/14659890500114359

[ref45] Griffiths M. (2009). Internet gambling in the workplace. J. Work. Learn. 21, 658–670. doi: 10.1108/13665620910996197

[ref46] Grinols E. L. (2011). The hidden social costs of gambling. Available online at: https://www.senate.ga.gov/committees/Documents/HiddenCostsofGam.pdf (Accessed June 23rd, 2024).

[ref47] Hellumbråten Kristensen J. Trifunovic S. Strand J. Kraft Vistnes K. Syvertsen A. Zandi A.. (2022). A systematic literature review of studies on attitudes towards gambling using the attitudes towards gambling scale (ATGS). Int. Gambl. Stud. 23, 353–386. doi: 10.1080/14459795.2022.2143856

[ref48] Hing N. Nuske E. Tolchard B. Russell A. (2014). What influences the types of help that problem gamblers choose? A preliminary grounded theory model. Int. J. Ment. Heal. Addict. 13, 241–256. doi: 10.1007/s11469-014-9525-y, PMID: 40078838

[ref49] Hing N. Tiyce M. Holdsworth L. Nuske E. (2013). All in the family: help-seeking by significant others of problem gamblers. Int. J. Ment. Heal. Addict. 11, 396–408. doi: 10.1007/s11469-012-9423-0

[ref50] Huic A. Kranzelic V. Dodig Hundric D. Ricijas N. (2017). Who really wins? Efficacy of a Croatian youth gambling prevention program. J. Gambl. Stud. 33, 1011–1033. doi: 10.1007/s10899-017-9668-4, PMID: 28108811

[ref51] James R. J. E. O’Malley C. Tunney R. J. (2016). Why are some games more addictive than others: the effects of timing and payoff on perseverance in a slot machine game. Front. Psychol. 7:46. doi: 10.3389/fpsyg.2016.00046, PMID: 26869955 PMC4735408

[ref52] Jelić M. Huić A. Dinić T. (2013). “Stav o kockanju i aktivnosti kockanja studenata” in Suvremeni izazovi psihologije rada i organizacijske psihologije: knjiga sažetaka. eds. Sušanj Z. Miletić I. Kalebić Maglica B. Lopižić J. (Zagreb: Hrvatsko psihološko društvo), 54.

[ref53] Jiménez-Murcia S. Álvarez-Moya E. M. Granero R. Neus Aymami M. Gómez-Peña M. Jaurrieta N.. (2007). Cognitive–behavioral group treatment for pathological gambling: analysis of effectiveness and predictors of therapy outcome. Psychother. Res. 17, 544–552. doi: 10.1080/10503300601158822

[ref54] Kalischuk R. G. Nowatzki N. Cardwell K. Klein K. Solowoniuk J. (2006). Problem gambling and its impact on families: a literature review. Int. Gambl. Stud. 6, 31–60. doi: 10.1080/14459790600644176

[ref55] Kim H. Y. (2013). Statistical notes for clinical researchers: assessing normal distribution (2) using skewness and kurtosis. Restorat. Dentist. Endodont. 38, 52–54. doi: 10.5395/rde.2013.38.1.52, PMID: 23495371 PMC3591587

[ref56] Kourgiantakis T. Saint-Jacques M.-C. Tremblay J. (2018). Facilitators and barriers to family involvement in problem gambling treatment. Int. J. Ment. Heal. Addict. 16, 291–312. doi: 10.1007/s11469-017-9742-2

[ref57] Ladouceur R. Sylvain C. Boutin C. Lachance S. Doucet C. Leblond J. (2003). Group therapy for pathological gamblers: a cognitive approach. Behav. Res. Ther. 41, 587–596. doi: 10.1016/s0005-7967(02)00036-0, PMID: 12711266

[ref58] Ladouceur R. Sylvain C. Boutin C. Lachance S. Doucet C. Leblond J.. (2001). Cognitive treatment of pathological gambling. J. Nerv. Ment. Dis. 189, 774–780. doi: 10.1097/00005053-200111000-00007, PMID: 11758661

[ref59] Landon J. Grayson E. Roberts A. (2018). An exploratory study of the impacts of gambling on affected others accessing a social service. Int. J. Ment. Heal. Addict. 16, 573–587. doi: 10.1007/s11469-017-9785-4

[ref60] Latvala T. Lintonen T. Konu A. (2019). Public health effects of gambling – debate on a conceptual model. BMC Public Health 19, 1077–1016. doi: 10.1186/s12889-019-7391-z, PMID: 31399026 PMC6688345

[ref61] Ledgerwood D. M. Loree A. M. Lundahl L. H. (2013). “Predictors of treatment outcome in disordered gambling” in The Wiley-Blackwell handbook of disordered gambling, Wiley Online. 283–305.

[ref9004] Lorains F. K. Cowlishaw S. Thomas S. A. (2011). Prevalence of comorbid disorders in problem and pathological gambling: systematic review and meta-analysis of population surveys. Addiction, 106, pp.490–498. doi: 10.1111/j.1360-0443.2010.03300.x 21210880

[ref62] Lovibond P. F. Lovibond S. H. (1995). The structure of negative emotional states: comparison of the depression anxiety stress scales (DASS) with the beck depression and anxiety inventories. Behav. Res. Ther. 33, 335–343. doi: 10.1016/0005-7967(94)00075-u, PMID: 7726811

[ref63] Makarovič M. Macur M. Rončević B. (2011). Policy challenges of problem gambling in Slovenia. Ljetopis socijalnog rada 18, 127–152.

[ref64] May R. K. Whelan J. P. Meyers A. W. Steenbergh T. A. (2005). Gambling-related irrational beliefs in the maintenance and modification of gambling behaviour. Int. Gambl. Stud. 5, 155–167. doi: 10.1080/14459790500303147

[ref65] Messerlian C. Derevensky J. Gupta R. (2005). Youth gambling problems: a public health perspective. Health Promot. Int. 20, 69–79. doi: 10.1093/heapro/dah509, PMID: 15681591

[ref66] Meyer G. Kalke J. Hayer T. (2018). The impact of supply reduction on the prevalence of gambling participation and disordered gambling behavior: a systematic review. SUCHT 64, 283–293. doi: 10.1024/0939-5911/a000562

[ref67] Mide M. Arvidson E. Söderpalm Gordh A. (2023). Clinical differences of mild, moderate, and severe gambling disorder in a sample of treatment seeking pathological gamblers in Sweden. J. Gambl. Stud. 39, 1129–1153. doi: 10.1007/s10899-022-10183-x, PMID: 36609904 PMC10397119

[ref68] Nation M. Crusto C. Wandersman A. Kumpfer K. L. Seybolt D. Morrissey-Kane E.. (2003). What works in prevention: principles of effective prevention programs. Am. Psychol. 58, 449–456. doi: 10.1037/0003-066x.58.6-7.449, PMID: 12971191

[ref69] Newall P. W. S. Moodie C. Reith G. Stead M. Critchlow N. Morgan A.. (2019). Gambling marketing from 2014 to 2018: a literature review. Curr. Addict. Rep. 6, 49–56. doi: 10.1007/s40429-019-00239-1

[ref70] Orford J. Griffiths M. Wardle H. Sproston K. Erens B. (2009). Negative public attitudes towards gambling: findings from the 2007 British gambling prevalence survey using a new attitude scale. Int. Gambl. Stud. 9, 39–54. doi: 10.1080/14459790802652217

[ref71] Orford J. Wardle H. Griffiths M. Sproston K. Erens B. (2010). The role of social factors in gambling: evidence from the 2007 British gambling prevalence survey. Community Work Fam. 13, 257–271. doi: 10.1080/13668803.2010.488101

[ref72] Petry N. M. (2005). Gamblers anonymous and cognitive-behavioral therapies for pathological gamblers. J. Gambl. Stud. 21, 27–33. doi: 10.1007/s10899-004-1919-5, PMID: 15789187

[ref73] Petry N. M. Ammerman Y. Bohl J. Doersch A. Gay H. Kadden R.. (2006). Cognitive-behavioral therapy for pathological gamblers. J. Consult. Clin. Psychol. 74, 555–567. doi: 10.1037/0022-006x.74.3.555, PMID: 16822112

[ref74] Petry N. M. Ginley M. K. Rash C. J. (2017). A systematic review of treatments for problem gambling. Psychol. Addict. Behav. 31, 951–961. doi: 10.1037/adb0000290, PMID: 28639817 PMC5714688

[ref75] Petry N. M. Litt M. D. Kadden R. Ledgerwood D. M. (2007). Do coping skills mediate the relationship between cognitive-behavioral therapy and reductions in gambling in pathological gamblers? Addiction 102, 1280–1291. doi: 10.1111/j.1360-0443.2007.01907.x, PMID: 17624978

[ref76] Petz B. Kolesarić V. Ivanec D. Milas G. Podlesek A. Galić Z. (2012). Petzova statistika: osnovne statističke metode za nematematičare. Jastrebarsko: Naklada Slap.

[ref77] Prochaska J. O. DiClemente C. C. (1983). Stages and processes of self-change of smoking: toward an integrative model of change. J. Consult. Clin. Psychol. 51, 390–395. doi: 10.1037/0022-006X.51.3.390, PMID: 6863699

[ref78] Prochaska J. O. DiClemente C. C. (1992). “The transtheoretical approach” in Handbook of psychotherapy integration. eds. Norcross J. C. Goldfried M. R. (Oxford: Oxford University Press Basic Books), 300–334.

[ref9008] Prochaska J. O. DiClemente C. C. (1986). Toward a comprehensive model of change, in Treating addictive behaviors: Processes of change, eds. Miller W. R. Heather N. (Boston, MA: Springer, US), pp. 3–27. Available at: https://link.springer.com/chapter/10.1007/978-1-4613-2191-0_1

[ref79] Rash C. Petry N. (2014). Psychological treatments for gambling disorder. Psychol. Res. Behav. Manag. 7, 285–295. doi: 10.2147/prbm.s40883, PMID: 25328420 PMC4199649

[ref80] Raspor A. Bulatović I. Stranjančević A. Lacmanović D. (2019). How important is gambling in national GDP: case study from Austria, Croatia, Italy and Slovenia. Economics 7, 31–49. doi: 10.2478/eoik-2019-0004, PMID: 39513035

[ref81] Razavi T. (2001). Self-report measures: an overview of concerns and limitations of questionnaire use in occupational stress research. Available online at: https://eprints.soton.ac.uk/35712/ (Accessed June 23rd, 2024).

[ref82] Ricijaš N. (2020). Mladi s problemima kockanja – koliko kockaju i kako vide industriju igara na sreću. Klinička psihologija 13, 47–62. doi: 10.21465/2020-kp-1-2-0004

[ref83] Ricijaš N. Dodig D. Huić A. Kranželić V. (2011). Navike i obilježja kockanja adolescenata u urbanim sredinama - izvještaj o rezultatima istraživanja. Available online at: https://www.croris.hr/crosbi/publikacija/rad-ostalo/772972 (Accessed November 27, 2024).

[ref84] Ricijaš N. Maglica T. Dodig Hundrić D. (2020). Regulativa igara na sreću u Hrvatskoj kao socijalni rizik. Ann. Soc. Work 26, 335–361. doi: 10.3935/ljsr.v26i3.297

[ref85] Rizeanu S. (2015). Pathological gambling treatment - review. Procedia. Soc. Behav. Sci. 187, 613–618. doi: 10.1016/j.sbspro.2015.03.114

[ref86] Sayegh C. S. Huey S. J. Zara E. J. Jhaveri K. (2017). Follow-up treatment effects of contingency management and motivational interviewing on substance use: a meta-analysis. Psychol. Addict. Behav. 31, 403–414. doi: 10.1037/adb0000277, PMID: 28437121

[ref87] Schwarzer R. Jerusalem M. (1995). “Generalized self-efficacy scale, in *measures in health psychology: a user’s portfolio*” in Causal and control beliefs. eds. Weinman J. Wright S. Johnston M. (Windsor: Nfer-Nelson), 35–37.

[ref88] Šimović H. Bajo A. Davidović M. Jelavić F. (2019). Gambling market in Croatia: financial performance and fiscal effect. Fiscus 4, 1–32. doi: 10.3326/efiscus.2019.9

[ref89] Skelin M. Puljić A. (2022). Gambling during the Covid-19 pandemic. Eur. Psychiatry 65, S128–S129. doi: 10.1192/j.eurpsy.2022.352

[ref90] Smedslund G. Berg R. C. Hammerstrøm K. T. Steiro A. Leiknes K. A. Dahl H. M.. (2011). Motivational interviewing for substance abuse. Campbell Syst. Rev. 7, 1–126. doi: 10.4073/csr.2011.6 PMC8939890 21563163

[ref91] Starcevic Kaic K. Ricijas N. Rojnic Palavra I. (2020). Standardi pružanja usluga psihosocijalnih intervencija Mreže za mlade: Mreža za mlade Grada Zagreba. Zagreb, Croatia.

[ref92] Steenbergh T. A. Meyers A. W. May R. K. Whelan J. P. (2002). Development and validation of the gamblers’ beliefs questionnaire. Psychol. Addict. Behav. 16, 143–149. doi: 10.1037/0893-164x.16.2.143, PMID: 12079253

[ref93] Stojnić D. (2018). Samoprocjena patoloških kockara o učinku psihosocijalnog tretmana u klubu ovisnika o kockanju. [PhD thesis]. Available online at: https://repozitorij.sfzg.unizg.hr/islandora/object/sfzg:532 (Accessed November 27, 2024).

[ref94] Stoltenberg S. F. Batien B. D. Birgenheir D. G. (2008). Does gender moderate associations among impulsivity and health-risk behaviors? Addict. Behav. 33, 252–265. doi: 10.1016/j.addbeh.2007.09.004, PMID: 17913380 PMC2225595

[ref95] Sulkunen P. Babor T. F. Cisneros Örnberg J. Egerer M. Hellman M. Livingstone C.. (2020). Setting limits: gambling, science and public policy—summary of results. Addiction 116, 32–40. doi: 10.1111/add.15241, PMID: 33084199

[ref96] Sylvain C. Ladouceur R. Boisvert J.-M. (1997). Cognitive and behavioral treatment of pathological gambling: a controlled study. J. Consult. Clin. Psychol. 65, 727–732. doi: 10.1037/0022-006x.65.5.727 9337491

[ref9005] Sussman S. Lisha N. Griffiths M. (2010). Prevalence of the Addictions: A Problem of the Majority or the Minority? Evaluation & the Health Professions, 34, pp.3–56. doi: 10.1177/0163278710380124 20876085 PMC3134413

[ref97] Toneatto T. Ladoceur R. (2003). Treatment of pathological gambling: a critical review of the literature. Psychol. Addict. Behav. 17, 284–292. doi: 10.1037/0893-164x.17.4.284, PMID: 14640824

[ref98] Uttl B. Kibreab M. (2011). Self-report measures of prospective memory are reliable but not valid. Can. J. Exp. Psychol. 65, 57–68. doi: 10.1037/a0022843 21443331

[ref99] van de Mortel T. F. (2008). Faking it: social desirability response bias in self-report research. Aust. J. Adv. Nurs. 25, 40–48.

[ref100] Welte J. W. Barnes G. M. Wieczorek W. F. Tidwell M. C. O. Parker J. C. (2004). Risk factors for pathological gambling. Addict. Behav. 29, 323–335. doi: 10.1016/j.addbeh.2003.08.007, PMID: 14732420

[ref101] WHO (2020). Addictive behaviour. Available online at: https://www.who.int/health-topics/addictive-behaviour#tab=tab_2 (Accessed June 23rd, 2024).

[ref102] Winters K. C. Derevensky J. L. (2019). A review of sports wagering: prevalence, characteristics of sports bettors, and association with problem gambling. J. Gambl. Iss. 43, 102–127. doi: 10.4309/jgi.2019.43.7, PMID: 29777460

[ref103] Yakovenko I. Quigley L. Hemmelgarn B. R. Hodgins D. C. Ronksley P. (2015). The efficacy of motivational interviewing for disordered gambling: systematic review and meta-analysis. Addict. Behav. 43, 72–82. doi: 10.1016/j.addbeh.2014.12.011, PMID: 25577724

[ref104] Zhou X. L. Goernert P. N. Corenblum B. (2018). Examining the efficacy of the GameSense gambling prevention programme among university undergraduate students. Int. Gambl. Stud. 19, 282–295. doi: 10.1080/14459795.2018.1554083, PMID: 40070530

[ref105] Zimet G. D. Dahlem N. W. Zimet S. G. Farley G. K. (1988). The multidimensional scale of perceived social support. J. Pers. Assess. 52, 30–41. doi: 10.1207/s15327752jpa5201_2 2280326

